# Knowledge, attitude and practice of insulin therapy in type 2 diabetes: Association with HbA1c

**DOI:** 10.6026/973206300221982

**Published:** 2026-04-30

**Authors:** Simhadri Lakshmi Roja, Priyanka Belur, Anirudha Deoke, Bezalel Kallukattil Binoy

**Affiliations:** 1 Department of Medicine, Ysbyty Ystrad Fawr, Aneurin Bevan University Health Board, Wales, United Kingdom; 2 Department of Emergency Medicine, JSS Hospital, Mysore, Karnataka, India; 3 Department of Community Medicine, NKP Salve Institute of Medical sciences & RC and LMH, Nagpur, India; 4 Department of Acute Medicine, Harrogate NHS Foundation Trust, North Yorkshire, United Kingdom

**Keywords:** Type 2 diabetes mellitus (T2DM), knowledge-attitude-practice (KAP), HbA1c, high-performance liquid chromatography

## Abstract

Poor knowledge, attitudes and practices regarding insulin therapy remain a major barrier to optimal glycemic control in patients with type 2 diabetes mellitus. This cross-sectional questionnaire study assessed 120 adults with T2DM to evaluate their understanding, beliefs and practices related to insulin use and its association with HbA1c. The mean HbA1c was 8.1% ± 1.4%, with a significant inverse correlation between total KAP scores and HbA1c levels (r = -0.42, p < 0.05). Although patients demonstrated moderate overall knowledge, gaps were identified in insulin administration techniques and related practices. Hence, the need for patient-centered education to address barriers and improve adherence, for optimizing long-term glycemic outcomes is reported.

## Background:

More than 90% of cases of global diabetes have been classified as type 2 diabetes mellitus (T2DM). The continuing increase in prevalence for T2DM worldwide is due to the association between persistent hyperglycaemia and microvascular and macrovascular complications [1]. It is the leading cause of morbidity and mortality, even though insulin therapy should be initiated in every patient whose glycaemic targets are not achieved by means of oral agents, including metformin [2]. However, the initiation and continuing adherence to insulin therapy is less than optimal in a majority of the patients [3]. Contributing to the sub-optimal adherence may be a psychological resistance to insulin, fear of injections, perceived dependence on the medications and lack of self-management skills [4]. Studies reveal that incorrect injection techniques, improper storage of insulin and inconsistent monitoring of blood glucose levels are frequent occurrences among insulin-using patients, even after many years of administering insulin [5]. These behavioral gaps have a direct impact on glycaemic control, as HbA1c levels reflect cumulative glucose levels over the previous two to three months and are the standard measure for assessing the efficacy of diabetes treatments [6]. Knowledge-Attitude-Practice (KAP) frameworks provide a platform for assessing the diabetes-related knowledge, attitudes and self-care behaviors of patients with diabetes [7]. Several recent studies have demonstrated a positive association between increased diabetes- related knowledge and a positive attitude toward medications with improved adherence and lower HbA1c levels [8]. Nevertheless, the majority of studies conducted to date have considered either knowledge or adherence status in isolation without looking at the relationship between all three KAP domains, along with objective levels of HbA1c, in populations of insulin-treated patients [9]. Recognizing how the combined KAP domains of patients with diabetes relate to glycaemic control may help identify modifiable factors that contribute to poor glycaemic control [10]. Therefore, it is of interest to evaluate the KAP of people with T2DM on the use of insulin therapy and its association with HbA1c levels in the population of a tertiary care hospital.

## Materials and Methods:

The purpose of this study was to evaluate the knowledge, attitude and practice of insulin therapy among type 2 diabetic patients who were treated with insulin for at least six months and had received treatment through a diabetes outpatient department at a tertiary care hospital. A total of 120 (including both men and women) adult patients with type 2 diabetes who had consented to participate in the study were enrolled. Patients with the following conditions were excluded from the study: type 1 diabetes, gestational diabetes, renal disease, liver disease, or mental illness. Ethics committee approval was obtained from the institution prior to starting the study. The demographic variables obtained during the study included age, sex, length of diabetes, educational level and length of time on insulin. A validated 25- item Knowledge, Attitude and Practice (KAP) tool specific to insulin therapy was used to collect information about how well patients understood felt and acted regarding their use of insulin therapy. The 25-item KAP tool consisted of three components: KAP-knowledge (10 items), KAP-attitude (8 items) and KAP-Practice (7 items). Patients received a score of 1 for each correct answer (or for a positive attitude) and a score of 0 for every incorrect answer (or a negative attitude). The total KAP scores were calculated and categorized as poor, average, or good based on predetermined cut-off scores. In addition to KAP, the study examined the degree of glycaemic control achieved by measuring glycated haemoglobin (HbA1c). The HbA1c levels of the patients were analysed at the hospital's central laboratory using high-performance liquid chromatography (HPLC) and reported as the (mean±SD). Demographic variables and KAP categories of patients were evaluated using descriptive statistics. The Pearson's Coefficient of Correlation was used to examine the relationship between the KAP total score and HbA1c for each patient. The means of HbA1c for each of the KAP-Knowledge and KAP-Attitude categories were compared using One-Way ANOVA. A p value of < 0.05 was considered statistically significant. All statistical analyses were performed using SPSS version 26.0.

## Results:

The study surveyed a sample of 120 adult patients diagnosed with Type 2 diabetes and receiving insulin therapy. Participants had a mean age of (56.7 years ± 9.3 years) and 56.7% of the participants were male. The average disease duration was (8.5 years ± 4.2 years), with a mean HbA1c of (8.1% ± 1.4%), indicating a lack of optimal glycaemic control. The mean total KAP score for all participants was (14.2 ± 3.6), which suggests some degree of overall awareness; nevertheless, participants exhibited major knowledge deficits across several areas. Approximately 32% of participants displayed good knowledge, 45% had a positive attitude toward diabetes management and correct practice levels ranged from 40% to 58%. There was a statistically significant negative correlation identified between the total KAP score and HbA1c (r = -0.42; p <0.05). Participants who exhibited good knowledge and positive attitudes had a significantly lower HbA1c than participants who fell into the poor knowledge attitude category. Table 1 (see PDF) (means, standard deviations) Mean Age (mean): 56.7 ± 9.3 and Male (56.7% male), Duration of Diabetes (mean): 8.5 ± 4.2 years, HbA1c Level (mean): 8.1 ± 1.4% (indicates suboptimal glycaemic control). Table 2 (see PDF) (good knowledge of insulin use): 32% of participants had Good Knowledge of Insulin Use; 48% had Average Knowledge; and 20% had Poor Knowledge. Table 3 (see PDF) (attitudes towards insulin therapy): 45% of participants had Positive Attitudes towards Insulin Therapy; 35% were Neutral; and 20% were Negative. Table 4 (see PDF) (self-reported Insulin Injection technique): 58% correctly identified the correct Injection Technique; 50% correctly stored Insulin; and 40% regularly rotated Injection Sites. Table 5 (see PDF) (glycaemic control): Mean HbA1c Levels increased progressively across KAP Knowledge Categories (Good Knowledge Group: Mean HbA1c 7.4%; Poor Knowledge Group: Mean HbA1c 8.9%). Table 6 (see PDF) (Relationship between HbA1c and Participants' Attitude towards Insulin Therapy): On average, participants with Positive Attitudes were less likely to maintain High HbA1c Levels (Mean HbA1c 7.5%) than participants with Neutral Attitudes (Mean HbA1c 8.4%) or Negative Attitudes towards Insulin Therapy (Mean HbA1c 9.0%). Table 7 (see PDF) demonstrates a significant inverse relationship between total KAP Score and HbA1c Levels (r = -0.42, p < 0.05); i.e., higher cumulative KAP Scores are associated with Better Glycaemic Control.

## Discussion:

This investigation shows that people with type 2 diabetes receiving insulin have poor knowledge, attitudes and practices regarding their insulin therapy, which is significantly correlated to their blood glucose control. The average HbA1c of 8.1% represents a high percentage of subjects with poor metabolic control [11]. Approximately one-fifth of the respondents had good levels of knowledge, while many of them also had significant defect in injecting technique (i.e., did not rotate injection sites) and proper storage for insulin. The inverse relationship between total KAP scores and HbA1c levels (Pearson's r = -0.42) indicates that behaviour and perception impact metabolic health directly [12]. Although respondents had diabetes for an average of just over eight years, there were still significant knowledge gaps. The amount of time that a person has been on insulin alone does not lead to an adequate level of understanding or proper technique for using insulin [13]. There is emerging evidence that structured education sessions at repeated intervals, rather than repeated passive professional exposure to insulin therapy, are necessary to achieve a high level of self-care with insulin [1, 2]. The increasing HbA1c means across different knowledge levels illustrates that there is a true gradient between knowledge and blood sugar levels [14]. Attitude about insulin is also critical to achieving good metabolic health outcomes. People with positive attitudes (perceptions) of insulin therapy had lower average HbA1c levels than those with neutral and/or negative attitudes. Insulin resistance that results from psychological causes is a major factor in the inability of people receiving insulin therapy to get the best results from their insulin therapy; fear of needles, stigma and misconceptions about dependency have been documented as common psychological barriers [3, 4]. Even when respondents had a good level of KAP, these attitudinal barriers may have still impaired their ability to adhere to their diabetes management plan. Our findings further emphasise that positive perceptions of insulin therapy are independent risk factors for improved blood glucose control [15]. There were also significant deficiencies in practice regarding the use of insulin primarily relating to the lack of proper site rotation and proper storing of insulin. The inability to correctly measure insulin may cause a decrease in the consistency of insulin absorption and contribute to wide fluctuations in blood glucose levels. Present day diabetes care models advocate competency-based skill retraining during follow up appointments versus single instructions at the time of initiation of insulin therapy [5, 6]. The KAP scores overall were moderate at best. Thus, as previously noted, practical training must have ongoing reinforcement. Correlating the structured KAP assessments to HbA1c changes provides a clearer picture of how cognitive (knowledge) and behavioural (practice) determinants relate to metabolic outcomes. Many previous studies have examined adherence to insulin alone or KAP independently. However, integrating the three forms of the KAP with objective data provides a comprehensive assessment of the relationship between behaviour and health [1, 2-3]. The data show that increased KAP increases results in lower HbA1c, validating the clinical relevance of education on improving metabolic health outcomes. These findings have a number of significant implications for the practice of diabetes care. Routine KAP assessment related to insulin therapy during outpatient appointments may help identify and develop an action plan around modifiable obstacles to attaining optimal metabolic control before the development of poor metabolic health. Cultural tailored and structured education regarding proper injection site rotation and storages practices, along with psychological counselling, may help improve adherence and ultimately decrease the risk of long-term complications of diabetes [7, 8]. Incorporating KAP evaluations into routine diabetes management will therefore provide an additional method for enhancing patient-centred diabetes care while improving blood glucose control beyond medication therapy alone. Some limitations of this study include the fact that it was a cross-sectional study and therefore results cannot be assumed to be causative in nature. Self-report bias could have affected the reliability of practice as reported. Furthermore, as the data were generated from one site, the generalizability of the network findings is limited. Longer-term studies that evaluate specific KAP interventions aimed at reducing HbA1c levels are necessary. KAP regarding insulin therapy remains an important determinant of blood glucose control and should be emphasised as a part of routine diabetes management plans.

## Conclusion:

Knowledge, attitudes and practical skills related to insulin therapy are significantly associated with glycaemic control in adults with type 2 diabetes. Moderate KAP levels and persistent technique deficiencies correspond with higher HbA1c values, highlighting modifiable behavioural determinants of poor metabolic outcomes. Hence, routine structured education and periodic behavioural assessment should be integrated into standard diabetes care to improve long-term glycaemic control.
